# Access to Mental Health Treatment Services in Asian Languages

**DOI:** 10.1001/jamahealthforum.2025.6858

**Published:** 2026-02-27

**Authors:** Aarya Suryavanshi, Jonathan Cantor, Sugy Choi, Ji Eun Chang

**Affiliations:** 1 RAND, Santa Monica, California; 2 New York University Grossman School of Medicine, New York; 3 Editorial Fellow, *JAMA Health Forum*; 4 New York University School of Global Public Health, New York

## Abstract

**Importance:**

Asian language speakers with limited English proficiency (LEP) face significant barriers to accessing adequate mental health care. Despite worsening mental health outcomes for this population, there is limited research examining the availability of Asian language mental health treatment in the US.

**Objective:**

To quantify trends and analyze disparities in the geographic availability of Asian language mental health treatment from 2015 to 2024.

**Design, Setting, and Participants:**

This cross-sectional study of US mental health facilities from April 30, 2015, to December 9, 2024, used longitudinal data from the nationally representative Mental Health and Addiction Treatment Tracking Repository linked with county-level demographic data from the 2023 American Community Survey. Facilities were included if they completed the National Mental Health Services Survey or the National Substance Use and Mental Health Services Survey.

**Main Outcomes and Measures:**

Primary outcomes included the annual proportion of mental health facilities offering Asian language services and the proportion of counties with at least 1 such facility. For 2024, facility-level characteristics associated with Asian language services were assessed and geographic mismatches between service availability and the proportion of Asian language–speaking individuals with LEP were mapped.

**Results:**

The study included 3847 mental health facilities. Of these, 214 facilities (5.6%) offered services in at least 1 Asian language in 2024 (including Arabic, Chinese, Farsi, Hindi, Hmong, Japanese, Korean, Tagolog, and Vietnamese). The proportion peaked at 265 facilities (6.9%) in 2021, then declined from 2022 to 2024. The number of counties with at least 1 facility with Asian language services was 98 (6.3%) in 2024. Facilities offering Asian language services were concentrated in metropolitan areas (208 [97.2%]), particularly in California (57 [26.6%]) and the Northeast (52 [24.3%]). Rural areas lacked such services (3 of 485 rural facilities [0.6%] in 2024), even in counties with substantial populations of Asian language–speaking individuals with LEP (0 of 5 facilities).

**Conclusions and Relevance:**

This cross-sectional study found a persistent geographic mismatch between the mental health needs of Asian language–speaking individuals with LEP and the availability of appropriate linguistic services. The gap was pronounced in rural areas. The findings suggest that policies aimed at expanding the behavioral health workforce and increasing access to culturally and linguistically competent services to reduce ongoing disparities in mental health outcomes and access to care are urgently needed.

## Key Points

**Question:**

Has the availability of mental health treatment services in Asian languages changed over time?

**Findings:**

In this cross-sectional study of 3847 US mental health facilities from 2015 to 2024, the availability of Asian language services remained persistently low and declined in later years, with limited access especially in rural counties.

**Meaning:**

The findings suggest that policies aimed at increasing access to culturally and linguistically competent mental health services are needed, especially in rural areas.

## Teaser

This cross-sectional study examines the availability of mental health treatment offered in Asian languages in US mental health facilities from 2015 to 2024.

## Introduction

More than 20% of adults in the US experience a mental health disorder, yet less than half receive treatment.^1,2^ This mental health treatment gap has both direct health costs and indirect economic costs.^1^ Rates of mental health service use are even lower among racial and ethnic minority populations, including Asian American individuals, who have some of the lowest rates of mental health service utilization despite increases in both the population of Asian immigrants in the US^3^ and mental health needs.^4^

The disproportionate mental health treatment gap is particularly alarming given that age-adjusted rates of suicide increased for Asian or Pacific Islander individuals between 2014 and 2019.^5^ The COVID-19 pandemic further worsened the mental health of Asian individuals compared with White individuals,^6,7,8,9,10,11^ partly due to heightened fears and experiences of discrimination and hate crimes.^12,13^

Asian American individuals have lower rates of mental health–related service use than the general population.^14^ Low-income Asian American individuals are among the populations least likely to visit any health care practitioner in a given year,^15^ and Asian American adolescents face additional barriers, such as low household income and lack of insurance.^16^ Stigma within Asian American communities associated with mental illness can lead to reduced reporting of psychiatric symptoms and reluctance to seek treatment.^17,18,19,20^ Language barriers and limited English proficiency (LEP) are also important obstacles to seeking and delivering appropriate care.^21,22,23,24^ Taken together, these factors help explain the persistently wide mental health treatment gap for the Asian American population.

Most studies on language services at mental health treatment facilities have focused on Spanish language accessibility,^25^ leaving a critical gap in understanding how Asian language services are distributed across the US.^26,27,28,29^ To our knowledge, no studies have examined changes over time in the availability of Asian language services at mental health treatment facilities or have linked longitudinal facilities measures to Asian language–speaking populations with LEP.^30,31^

These gaps emerge despite long-standing federal language access policies. Executive Order 13166, the US Department of Health and Human Services Office for Civil Rights’ guidance on LEP, and the National Culturally and Linguistically Appropriate Services (CLAS) Standards require federally funded health care organizations to ensure meaningful access to their services for individuals with LEP.^30,32,33^ These policies, along with policy statements from professional organizations such as the American Public Health Association, underscore the expectation that behavioral health services be linguistically accessible.^34^

In this study, we used national data from April 30, 2015, to December 9, 2024, to examine trends in the availability of mental health treatment services offered in Asian languages, the geographic distribution of these facilities, and whether availability aligned with potential local population need, defined as the proportion of Asian language–speaking residents with LEP residing in a given county. While our primary focus was on Asian languages, this study reflects broader commitments outlined in the CLAS Standards and federal language access requirements, which emphasize equitable communication.^30,31^

## Methods

### Data

This cross-sectional study used data from the Mental Health and Addiction Treatment Tracking Repository (MATTR), which compiles annual versions of the National Directory of Mental Health Treatment Facilities from the Substance Abuse and Mental Health Services Administration (SAMHSA). These directories are based on responses to the SAMHSA National Mental Health Services Survey (N-MHSS) and the National Substance Use and Mental Health Services Survey (N-SUMHSS) and include only licensed facilities that agree to be listed. The National Directory of Mental Health Treatment Facilities and MATTR do not include all N-SUMHSS items; for example, we could not distinguish whether treatment services were delivered by on-site counselors or on-call interpreters. We followed the Strengthening the Reporting of Observational Studies in Epidemiology (STROBE) reporting guideline for cross-sectional studies. This study did not involve human participant research as defined by 45 CFR §46 and was deemed exempt by the RAND Human Subject Protection Committee.

SAMHSA began fielding the N-MHSS in 2008. In 2021, it merged with the National Survey of Substance Abuse Treatment Services to create the N-SUMHSS. Using geographic identifiers from MATTR, we linked the facility data to county-level demographic information from the American Community Survey (ACS). For this study, we used 5-year estimates from the 2023 ACS.

### Measures

From MATTR, we examined language services and select facility characteristics. For Asian language services, the N-SUMHSS asks whether a facility offers services in sign language (eg, American Sign Language, Signed English, or cued speech) and whether staff counselors provide treatment in languages other than English. Facilities that answer “yes” select languages from a list. Responses may reflect direct bilingual care or interpreter-supported services. Our indicator therefore reflects linguistically accessible services rather than strictly concordant care. For multiyear comparability, our analysis only included languages that were available for all years of MATTR from 2015 to 2024. A total of 22 languages were included on the survey in all years; 9 were Asian languages (Arabic, Chinese, Farsi, Hindi, Hmong, Japanese, Korean, Tagalog, and Vietnamese).

Facility characteristics used as covariates included self-reported facility ownership (public, private for-profit, or private nonprofit), accepted payment options (Medicaid, Medicare, self-pay, private insurance, or other), service setting (outpatient only or inpatient), and whether the facility was in a metropolitan county. Facility characteristics were analyzed using 2024 data only. Facilities with missing ownership information were grouped with publicly owned facilities.

We linked facilities to county-level demographic data from the 2023 ACS 5-year estimates. The ACS aggregates Asian and Pacific Islander languages into a single category. Because MATTR does not collect Pacific Islander languages, we used this ACS category as a proxy for residents who spoke Asian languages at home and English “less than very well.” In addition, we included residents who reported speaking an Other Indo-European language and speaking English “less than very well” because this category contains several South Asian languages (eg, Hindi, Urdu, and Bengali). Census documentation indicated that approximately 35% of these languages are South Asian languages, while the remaining 65% are non-Asian languages (eTable 1 in Supplement 1). Thus, our county-level measure captured a substantial share of Asian adults with LEP but also introduced measurement error.^35^

### Statistical Analysis

First, we conducted descriptive analyses to summarize facility characteristics for all mental health treatment facilities that were active in 2024 and subsets offering any non-English services (including American Sign Language) and Asian language services. We used multivariable logistic regression models to estimate whether a facility offered any non-English services and Asian language services, with standard errors clustered by state. We report the log odds ratios (ORs) with 95% CIs.

Next, we examined the percentage of facilities offering Asian language services and compared it with the percentage of facilities offering any non-English service from 2015 to 2024. We repeated this comparison for the percentage of counties with at least 1 facility offering the specified language service. Finally, we mapped the mismatch between the proportion of the population that spoke an Asian language with LEP and the number of mental health treatment facilities offering Asian language services using heat maps. Counties were grouped into 6 categories based on the proportion of Asian language speakers with LEP (0%, >0 to 1%, >1 to 2%, >2 to 5%, >5 to 10%, and >10%) for interpretability. All analyses were conducted in R, version 4.4.1 (R Foundation for Statistical Computing) and Stata, version 18.0 (Stata Corp LLC). Two-sided *P* < .05 was considered significant.

## Results

The data included 3847 facilities that appeared throughout the entire study period. In 2024, 3098 facilities (80.5%) offered treatment services with at least 1 non-English language and 214 facilities (5.6%) offered treatment services with at least 1 Asian language. Table 1 reports facility characteristics by language service. Compared with all facilities, those offering any non-English services were slightly less likely to be private for-profit and more likely to accept Medicare, Medicaid, self-pay, and private insurance. They were also somewhat more likely to be outpatient only, with a similar share located in metropolitan counties.

**Table 1.** Characteristics of Mental Health Facilities Categorized by the Language Services Provided in 2024

Characteristic	Facilities, No. (%)^a^		
	All (N = 3847)	With any non-English language service (n = 3098)^b^	With any Asian language service (n = 214)
Ownership^c^			
Public	780 (20.3)	667 (21.5)	48 (22.4)
Private for-profit	502 (13.0)	320 (10.3)	23 (10.7)
Private nonprofit	2562 (66.6)	2111 (68.1)	143 (66.8)
Payment type accepted^d^			
Medicare	3073 (79.9)	2619 (84.5)	177 (82.7)
Medicaid	3620 (94.1)	2955 (95.4)	195 (91.1)
Self-pay	3489 (90.7)	2860 (92.3)	179 (83.6)
Private insurance	3370 (87.6)	2773 (89.5)	167 (78.0)
Other	42 (1.1)	33 (1.1)	3 (1.4)
Missing	4 (0.1)	2 (0.1)	0
Outpatient setting only	2727 (70.9)	2254 (72.8)	149 (69.6)
Located within a metropolitan county	3252 (84.5)	2633 (85.0)	208 (97.2)

^a^ Facilities in the US and the District of Columbia that appeared in each year from 2015 to 2024.

^b^ Includes facilities offering Asian language services (reported languages included Arabic, Chinese, Farsi, Hindi, Hmong, Japanese, Korean, Tagolog, and Vietnamese).

^c^ Facilities with missing ownership (n = 2) were grouped with public facilities.

^d^ Categories are not mutually exclusive.

For facilities offering any Asian language treatment services, a higher proportion were publicly owned (48 of 214 [22.4%]) compared with facilities offering any non-English treatment services (667 of 3098 [21.5%]) and all facilities (780 of 3847 [20.3%]). Facilities offering any Asian language treatment services were less likely to be owned by a private for-profit company (23 [10.7%] vs 502 [13.0%]) and more likely to accept Medicare (177 [82.7%] vs 3073 [79.9%]). Facilities offering Asian language treatment services were less likely to accept Medicaid (195 [91.1%] vs 3620 [94.1%] vs 2955 [95.4%]), self-pay (179 [83.6%] vs 3489 [90.7%] vs 2860 [92.3%]), and private (167 [78.0%] vs 3370 [87.6%] vs 2773 [89.5%]) insurance for payment compared with all facilities and facilities offering a non-English treatment service, respectively. Facilities offering treatment services with an Asian language were also less likely to be outpatient only (149 [69.6%]) compared with all facilities (2727 [70.9%]) and facilities offering a non-English treatment service (2254 [72.8%]). However, a larger portion of facilities offering treatment services in an Asian language were in metropolitan counties (208 [97.2%]) compared with all facilities (3252 [84.5%]) or facilities offering treatment services in any non-English language (2633 [85.0%]).

Table 2 presents the results of multivariable logistic regression models estimating whether a facility offered non-English or Asian language mental health services in 2024. Compared with facilities that did not offer services in any non-English language, those that did were significantly less likely to be private for-profit (OR, 0.30; 95% CI, 0.20-0.45) and more likely to accept all types of insurance, including Medicare, Medicaid, private insurance, and self-pay. They also had higher odds of being outpatient-only facilities (OR, 1.29; 95% CI, 1.06-1.57). Facilities offering Asian language services specifically were more likely to accept Medicare (OR, 1.72; 95% CI, 1.10-2.70) and were substantially more likely to be located in metropolitan counties (OR, 4.74; 95% CI, 1.55-14.53) compared with facilities that did not offer any services in an Asian language. Sensitivity analyses disaggregating Asian language services into individual languages indicated that these associations were not driven by any single language (eTable 2 in Supplement 1).

**Table 2.** Multivariate Logistic Regression Estimating the Likelihood of Facilities Offering a Specified Language Service

Variable	Odds ratio (95% CI)^a^	
	Facilities offering treatment services in any non-English language (n = 3098)^b^	Facilities offering treatment services in Asian language (n = 214)
Ownership^c^		
Private for-profit	0.30 (0.20-0.45)^d^	0.89 (0.49-1.58)
Private nonprofit	0.73 (0.51-1.05)	1.10 (0.83-1.46)
Payment type accepted		
Medicaid	3.21 (2.06-4.98)^d^	0.85 (0.51-1.43)
Medicare	2.43 (1.85-3.20)^d^	1.72 (1.10-2.70)^e^
Self-pay	1.67 (1.05-2.65)^e^	0.99 (0.56-1.69)
Private insurance	1.79 (1.10-2.89)^e^	0.81 (0.46-1.41)
Other	6.78 (2.22-20.68)^d^	0.86 (0.21-3.50)
Outpatient setting only	1.29 (1.06-1.57)^d^	0.99 (0.70-1.38)
Located within a metropolitan county	1.31 (0.94-1.83)	4.74 (1.55-14.53)^d^

^a^ For each model, the reference group is facilities that did not offer the specified language service. All models adjusted for the listed facility characteristics, with standard errors clustered at the state level.

^b^ Includes facilities offering Asian languages (reported languages included Arabic, Chinese, Farsi, Hindi, Hmong, Japanese, Korean, Tagolog, and Vietnamese).

^c^ Public ownership was omitted.

^d^ *P* < .01.

^e^ *P* < .05.

**Figure 1.**
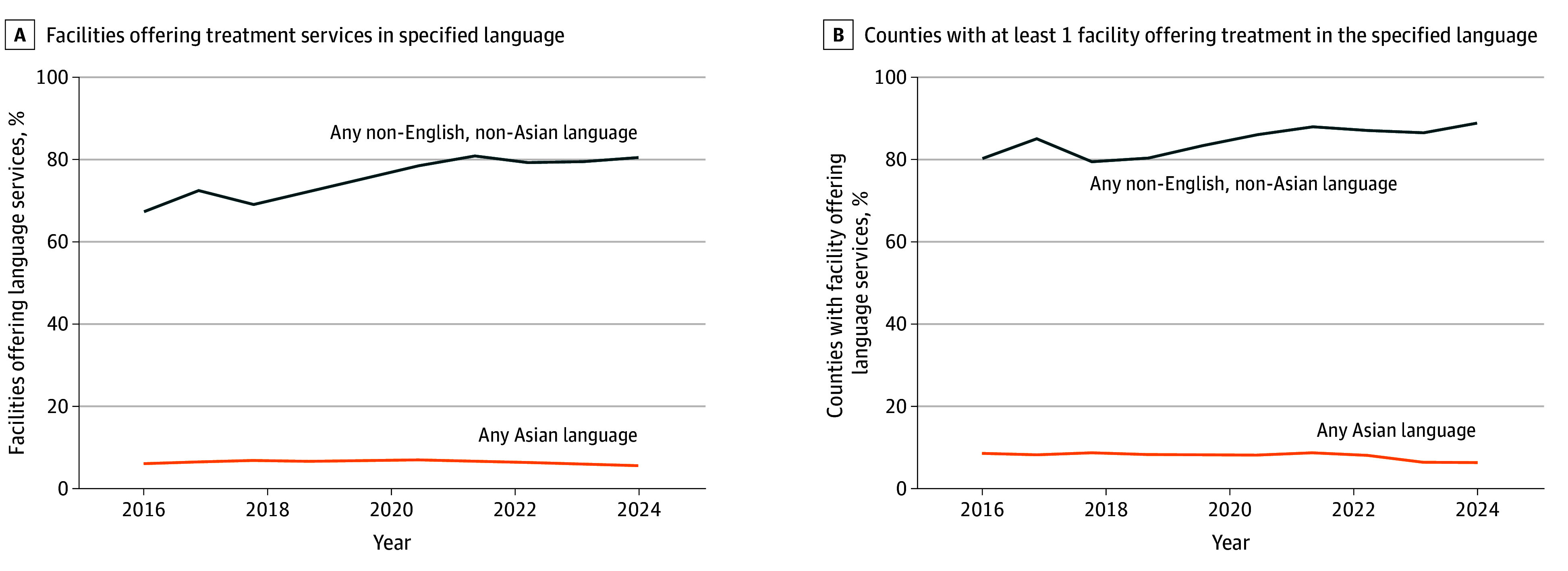
Line Graphs Showing the Percentage of All Facilities Offering Mental Health Treatment Services in a Specified Language and Counties With at Least 1 Facility That Offers Mental Health Treatment by Language From 2015 to 2024

Figure 1A shows trends in the proportion of mental health facilities offering treatment services in any non-English language (excluding Asian languages) compared with those offering services in an Asian language from 2015 to 2024. The share of facilities providing any non-English language services rose sharply in 2016 (2787 [72.4%]), peaked in 2021 (3102 [80.6%]), and then declined in 2022 (3050 [79.3%]) before beginning to rise again. By contrast, the proportion of facilities offering Asian language services remained relatively flat over time, with a modest peak in 2019 (263 [6.8%]), followed by a steady decline after 2021 (265 [6.9%]). The lowest levels of Asian language service availability were observed in 2023 (215 [5.6%]) and 2024 (214 [5.6%]).

Figure 1B illustrates trends in the proportion of US counties with at least 1 facility offering treatment services in a non-English or an Asian language from 2015 to 2024. The proportion of counties with a facility offering any non-English language services rose sharply in 2016 (1327 [85.0%]), continued increasing through 2021 (1373 [87.9%]), and then declined before rising again in 2024 (1391 [89.1%]). By contrast, the share of counties with a facility offering Asian language services remained relatively stable over time, peaking in 2017 (138 [8.8%]), but showing a gradual decline from 2021 (134 [8.6%]) to 2024 (98 [6.3%]).

**Figure 2.**
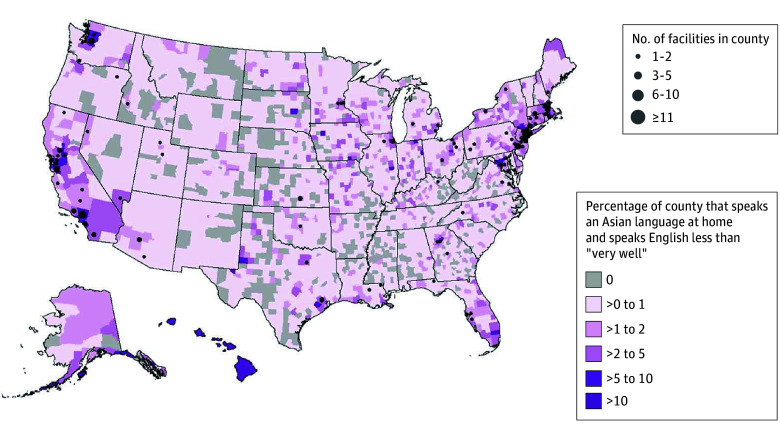
Map Showing the Number of Mental Health Facilities Offering Treatment in Any Asian Language and the County-Level Percentage of Asian Language–Speaking Individuals With Limited English Proficiency in 2024

Figure 2 shows a county-level mismatch in 2024 between the population of Asian language–speaking individuals with LEP and the availability of mental health facilities offering services in an Asian language. Facilities offering Asian language services were concentrated in metropolitan areas (208 [97.2%]), particularly in California (57 [26.6%]) and the Northeast (52 [24.3%]). Notably, 35 of 57 counties with a high proportion (>5%) of Asian language speakers with LEP (61.4%) had no facility offering treatment services in an Asian language. Rural areas lacked such services (3 of 485 rural facilities [0.6%] in 2024), even in counties with substantial populations of Asian language–speaking individuals with LEP (0 of 5 facilities), highlighting a significant geographic gap in access to linguistically appropriate care.

## Discussion

Asian individuals are the fastest growing racial or ethnic group in the US, with the population increasing by 81% between 2000 and 2019.^36^ In 2020, approximately 24 million people identified as Asian alone or in combination with another race—a number projected to more than double by 2060. Nearly 1 in 3 Asian American adults (30.8%) have LEP, meaning they do not speak English very well.^37,38^ Despite this rapid growth and substantial language need, our national analysis of mental health treatment facilities from 2015 to 2024 showed that behavioral health services in Asian languages remained limited and declined in recent years. The proportion of facilities offering Asian language services stayed relatively flat, with a noticeable decrease after 2020, even as the Asian American population faced disproportionate mental health burdens during the COVID-19 pandemic.^39,40^ These patterns highlight potential gaps in linguistically accessible services, likely reflecting a combination of workforce, geographic, and resource constraints.

This finding is critical, as patients with LEP may receive lower quality mental health care even with interpreters due to inadequate interpretation services.^41^ Qualitative research has shown that inadequate or ad hoc interpretation services may lead to symptoms and medical information getting lost in translation, leading to lower patient satisfaction and worse treatment outcomes.^42^ These patterns, together with prior research on interpreter limitations,^43,44^ underscore the rationale for direct language services provided by bilingual counselors and mental health treatment practitioners to bridge this gap.

Our study also found that rural areas lacked facilities offering Asian language services even in counties with comparatively large populations of Asian American individuals with LEP. This is especially salient because individuals living in rural areas report worse mental health outcomes than people living in urban areas.^45^ For Asian language–speaking individuals with LEP who live in rural areas, the challenges of their geographic location and language barrier may amplify one another, possibly contributing to poorer mental health outcomes. These compounding barriers indicate the importance of addressing geographic and language disparities in mental health care.

For many Asian language–speaking groups, particularly those with smaller populations or dispersed settlement patterns, it is neither feasible nor realistic to maintain a fully bilingual behavioral health workforce in every community, especially in rural areas.^46,47^ In these settings, interpreter services are not simply a supplement but a core component of language access.^48^ A practical policy response must balance efforts to diversify the mental health workforce with investments in high-quality interpreter infrastructure for Asian languages, ensuring understandable and culturally informed care even where bilingual services are scarce.^49^ Our study further highlights a dearth of adequate language services for Asian language–speaking individuals with LEP; this may exacerbate the existing mental health care gap and contribute to worsening mental health outcomes for this population.

Prior research^50,51^ has consistently identified language barriers as a major obstacle to behavioral health care access for individuals with LEP. Our findings support and extend this literature by documenting the limited and uneven distribution of Asian language services even in counties with substantial populations of Asian language–speaking individuals.^3^

Our study has several policy implications. First, federal and state initiatives should target the expansion of linguistically concordant behavioral health services, especially in underserved regions. Strategies could include incentivizing the hiring of multilingual health care practitioners, supporting interpreter infrastructure, or embedding virtual or artificial intelligence–enhanced translation services with appropriate quality controls. Second, workforce development efforts should prioritize linguistic and cultural competence tailored to the diverse subgroups within the Asian American population. Finally, improved data collection and public reporting on language offerings, especially disaggregated by specific Asian languages, are essential to guide resource allocation and policy design.^52,53^

These findings should be viewed in light of long-standing federal language access requirements, including Executive Order 13166,^32^ Title VI of the Civil Rights Act,^33^ and the National CLAS Standards.^30^ The limited availability of Asian language services suggests a gap between these mandates and current behavioral health infrastructure. Investments in interpreter systems, multilingual workforce capacity, and improved monitoring could help close this gap. The persistently low availability of Asian language services in mental health treatment settings suggests a misalignment between these mandates and the current behavioral health infrastructure. Strengthening interpreter infrastructure, supporting the hiring and retention of multilingual clinicians, and improving monitoring of language service availability may help bring practice closer to these standards.

It is also important to interpret these findings within both demand- and supply-side contexts. While cultural stigma and preferences for informal or community-based support may influence help-seeking behaviors among some Asian American subgroups, the geographic clustering of linguistically concordant services in metropolitan areas and their near absence in rural regions suggest supply and workforce distribution as major contributors to inequities in access to language-concordant behavioral health services. Addressing these disparities will require expanding the pipeline of multilingual behavioral health professionals, incentivizing placement in underserved areas, and supporting community-based organizations that provide linguistically and culturally tailored care.

This study provides a comprehensive national longitudinal and spatial analysis of mental health treatment facilities offering Asian language services. Our findings underscore an urgent need to strengthen the behavioral health infrastructure to meet the linguistic and cultural needs of a growing and diverse Asian American population.

### Limitations

This study has several limitations. First, our analysis of county-level potential language need relies on data from the ACS, which aggregates Asian and non-Asian languages under broad categories, limiting precision. The ACS combines Asian and Pacific Islander languages into a single category, which we used as a proxy for Asian language with LEP because MATTR does not include Pacific Islander languages. The Other Indo-European language category also mixes South Asian and non-Asian languages, introducing measurement error and highlighting the need for more granular language data. Second, the N-MHSS and N-SUMHSS are voluntary, self-reported surveys; despite high response rates among mental health care facilities (87.9%) during our study period, social desirability bias may influence reporting of language services. Third, we could not assess the quality or depth of language services; some facilities may report offering non-English language services even if no staff counselor speaks the language themselves, relying instead on interpreter services. As a result, our measure may overestimate the availability of linguistically concordant services. Fourth, MATTR lacks data on the racial and ethnic composition of practitioners and patients and does not capture the number of staff delivering treatment in languages other than English. Fifth, our period of study included the COVID-19 pandemic, and our findings have not been fully adjusted for the effects of the pandemic. Finally, while this study identified geographic analysis of linguistic mismatches that may underlie disparities in access, it did not directly link these service gaps to individual-level mental health outcomes or utilization patterns. Future research should integrate patient-level data to assess whether counties with limited language-concordant services are associated with worse access or outcomes among Asian American populations.

## Conclusions

This cross-sectional study found that despite increasing demand, behavioral health services in Asian languages remained limited in the US from 2015 to 2024 and availability declined in some areas. This limited access may contribute to structural barriers in accessing care for millions of Asian American individuals with LEP. Our findings suggest that policymakers and health systems should take action to ensure that behavioral health infrastructure supports the availability and sustainability of linguistically concordant services, recognizing workforce and resource constraints that affect service provision.

## Notes

Supplement 1. eTable 1. American Community Survey language classification for groups containing Asian languages eTable 2. Sensitivity analysis of individual Asian languages

Supplement 2. Data Sharing Statement
