# Supplementary material for: Access to Mental Health Treatment Services in Asian Languages
Source: JAMA Health Forum. 2026 Feb 27;7(2):e256858. doi: 10.1001/jamahealthforum.2025.6858 (PMC12949444; doi:10.1001/jamahealthforum.2025.6858)
Supplement: Supplement 1. — eTable 1. American Community Survey language classification for groups containing Asian languages eTable 2. Sensitivity analysis of individual Asian languages [file jamahealthforum-e256858-s001.pdf]

Supplemental Online Content

Suryavanshi A, Cantor J, Choi S, Chang JE. Access to mental health treatment services in Asian languages. *JAMA Health Forum*. 2026;7(2):e256858.
doi:10.1001/jamahealthforum.2025.6858

eTable 1. American Community Survey language classification for groups containing Asian languages

eTable 2. Sensitivity analysis of individual Asian languages

This supplemental material has been provided by the authors to give readers additional information about their work.

© 2026 Suryavanshi A et al. *JAMA Health Forum*.

eTable 1: American Community Survey language classification for groups containing Asian languages.

ACS Language group	***Forty-Two Group Classification***	***Asian Language***
Other Indo-European languages	French (incl. Cajun)	
	Haitian	
	Italian	
	Portuguese	
	German	
	Yiddish, Pennsylvania Dutch or other West Germanic languages	
	Greek	
	Russian	
	Polish	
	Serbo-Croatian	
	Ukrainian or other Slavic languages	
	Armenian	
	Persian (incl. Farsi, Dari)	Yes
	Gujarati	Yes
	Hindi	Yes
	Urdu	Yes
	Punjabi	Yes
	Bengali	Yes
	Nepali, Marathi, or other Indic languages	Yes
	Other Indo-European languages	
Asian and Pacific Island languages	Telugu	Yes
	Tamil	Yes

© 2026 Suryavanshi A et al. *JAMA Health Forum*.

Malayalam, Kannada, or other Dravidian languages	Yes
Chinese (incl. Mandarin, Cantonese)	Yes
Japanese	Yes
Korean	Yes
Hmong	Yes
Vietnamese	Yes
Khmer	Yes
Thai, Lao, or other Tai-Kadai languages	Yes
Other languages of Asia	Yes
Tagalog (incl. Filipino)	Yes
Ilocano, Samoan, Hawaiian, or other Austronesian languages	

Note: Table from United States Census Bureau. Final column added by authors to indicate Asian languages.

© 2026 Suryavanshi A et al. *JAMA Health Forum*.

eTable 2 Sensitivity analysis of individual Asian languages

	Facilities Offering Any Chinese Language *OR [95% CI]*	Facilities Offering Hindi *OR [95% CI]*	Facilities Offering Farsi *OR [95% CI]*	Facilities Offering Arabic *OR [95% CI]*	Facilities Offering Tagalog *OR [95% CI]*	Facilities Offering Korean *OR [95% CI]*
Ownership						
Public	*Base Category*	*Base Category*	*Base Category*	*Base Category*	*Base Category*	*Base Category*
Private for profit	1.05 [0.47, 2.33]	0.87 [0.36, 2.07]	1.31 [0.45, 3.81]	1.37 [0.52, 3.63]	0.73 [0.34, 1.59]	0.94 [0.35, 2.58]
Private non-profit	0.86, 0.39, 1.87]	0.92 [0.46, 1.82]	1.18 [0.60, 2.34]	1.12 [0.55, 2.31]	0.79 [0.42, 1.51]	0.69 [0.33, 1.35]
Forms of payment accepted						
Medicaid	[0.71, 0.39, 1.87]	2.04 [0.65, 6.43]	2.32 [1.03, 5.23]	1.95 [0.36, 10.70]	0.72 [0.26, 1.98]	1.23 [0.29, 5.22]
Medicare	1.60 [0.79, 3.25]	1.38 [0.71, 2.71]	2.82 [1.53, 5.20]**	2.08 [0.89, 4.87]	**4.46 [2.08, 9.58]****	1.46 [0.74, 2.89]
Self-Pay	0.72 [0.23, 2.21]	1.25 [0.69, 2.25]	1.05 [0.61, 1.79]	0.77 [0.30, 1.92]	0.69 [0.27, 1.75]	1.10 [0.46, 2.62]
Private	0.94 [0.37, 2.35]	1.21 [0.37, 2.79]	0.71 [0.43, 1.20]	0.90 [0.33, 2.44]	0.91 [0.53, 1.55]	0.82 [0.54, 1.26]
Other	0	2.09 [0.12, 35.71]	0	0	1.13 [0.28, 4.59]	0
Missing	NA	NA	NA	NA	NA	NA
Outpatient setting only	0.79 [0.54, 1.15]	0.72 [0.48, 1.09]	0.75 [0.48, 1.16]	**0.54 [0.30, 1.00]****	**0.41 [0.25, 0.69]****	0.63 [0.34, 1.18]
Located within a metropolitan county	**+**	+	+	+	+	+

Note: Sensitivity analysis was done on Asian languages that were offered in at least 10% of facilities that offered any Asian language in 2024. Of facilities that offered Asian languages in 2024, 16.2% offered Any Chinese Language, 15.0% offered Hindi, 12.5% offered Farsi,

© 2026 Suryavanshi A et al. *JAMA Health Forum*.

12.1% offered Arabic, 12.1% offered Tagalog, and 10.1% offered Korean. ** marks statistical significance at 0.01 level, * marks statistical significance at 0.05 level, and + indicates perfect or quasi-complete separation with an unstable estimate.

© 2026 Suryavanshi A et al. *JAMA Health Forum*.
