# Supplementary material for: Access to Mental Health Treatment Services in Asian Languages
Source: JAMA Health Forum. 2026 Feb 27;7(2):e256858. doi: 10.1001/jamahealthforum.2025.6858 (PMC12949444; doi:10.1001/jamahealthforum.2025.6858)
Supplement: Supplement 2. — Data Sharing Statement [file jamahealthforum-e256858-s002.pdf]

Data Sharing Statement

Suryavanshi. Access to Mental Health Treatment Services in Asian Languages. *JAMA Health Forum*. Published February 27, 2026. doi:10.1001/jamahealthforum.2025.6858

Data
Data available: Yes
Data types: Data (not involving human participants)
How to access data: jcantor@rand.org
When available: With publication

Supporting Documents
Document types: None

Additional Information
Who can access the data: researchers whose proposed use of the data has been approved
Types of analyses: for any purpose
Mechanisms of data availability: with approval of a proposal
